# Friend or Foe—Light Availability Determines the Relationship between Mycorrhizal Fungi, Rhizobia and Lima Bean (*Phaseolus lunatus* L.)

**DOI:** 10.1371/journal.pone.0154116

**Published:** 2016-05-02

**Authors:** Daniel J. Ballhorn, Martin Schädler, Jacob D. Elias, Jess A. Millar, Stefanie Kautz

**Affiliations:** 1 Department of Biology, Portland State University, Portland, Oregon, 97201, United States of America; 2 Helmholtz-Centre for Environmental Research, Dept. Community Ecology, 06120, Halle, Germany; 3 German Centre for Integrative Biodiversity Research Halle-Jena-Leipzig (iDiv), Deutscher Platz 5e, 04103, Leipzig, Germany; Estación Experimental del Zaidín (CSIC), SPAIN

## Abstract

Plant associations with root microbes represent some of the most important symbioses on earth. While often critically promoting plant fitness, nitrogen-fixing rhizobia and arbuscular mycorrhizal fungi (AMF) also demand significant carbohydrate allocation in exchange for key nutrients. Though plants may often compensate for carbon loss, constraints may arise under light limitation when plants cannot extensively increase photosynthesis. Under such conditions, costs for maintaining symbioses may outweigh benefits, turning mutualist microbes into parasites, resulting in reduced plant growth and reproduction. In natural systems plants commonly grow with different symbionts simultaneously which again may interact with each other. This might add complexity to the responses of such multipartite relationships. We experimented with lima bean (*Phaseolus lunatus*), which efficiently forms associations with both types of root symbionts. We applied full light and low-light to each of four treatments of microbial inoculation. After an incubation period of 14 weeks, we quantified vegetative aboveground and belowground biomass and number and viability of seeds to determine effects of combined inoculant and light treatment on plant fitness. Under light-limited conditions, vegetative and reproductive traits were inhibited in AMF and rhizobia inoculated lima bean plants relative to controls (un-colonized plants). Strikingly, reductions in seed production were most critical in combined treatments with rhizobia x AMF. Our findings suggest microbial root symbionts create additive costs resulting in decreased plant fitness under light-limited conditions.

## Introduction

Rhizobia and arbuscular mycorrhizal fungi (AMF) represent two major groups of plant-associated microbial mutualists [1–3]. Legume-associated rhizobia are nitrogen-fixing bacteria that play a key role for local and global nitrogen cycles, dramatically influencing the productivity and species composition of natural and agricultural ecosystems [2–4]. AMF colonize roots of the host plant, form extensive networks, and participate in the acquisition of nutrients (namely phosphorus) and water [1]. The association between plants and AMF is likely the most ubiquitous of all mutualisms, having been observed in 400 million year-old fossils and persisting in more than 80% of extant land plants [5]. Like rhizobia, AMF are considered keystone species in terrestrial ecosystems [1] due to their critical impact on plant growth and species composition [6].

Root colonization with rhizobia and AMF generally have positive effects on plant growth [2,7,8], including increases in vegetative and reproductive traits [9,10]. In legumes, which are frequently colonized by both types of microbial symbionts simultaneously, plant growth is usually enhanced by the dual symbiosis [11]. However, while most studies have been carried out under optimal conditions, in several common bean (*Phaseolus vulgaris*) varieties, under drought stress both dual and single colonization with different rhizobia strains and AM fungi can result in negative effects for the plant host [12]. Under such water limited conditions Franzini and co-workers [12] showed that AMF inhibited rhizobial nodule development and N_2_ fixation, and thus caused diminution of plant growth.

While costs of simultaneous colonization by rhizobia and AMF for their legume hosts have been reported under water limited conditions, less information exists on the effects of another key plant resource: light. Both rhizospheric associations, rhizobia and AMF, incur significant costs as consumers of plant photosynthates as the combined demand of symbionts can reach up to 28% of carbon fixed by the plant [13]. Plants can compensate for this cost through sink stimulation of photosynthesis, which is considered to be an adaptation to take advantage of nutrient supply enhancement by the symbiont without compromising the total amount of photosynthates available for plant functioning [13]. While sink stimulation is generally an efficient strategy to compensate for costs of carbohydrate allocation, the question arises of how plants respond to microbial inoculation when photosynthesis cannot easily be increased [14], for example under light-limited conditions [15,16]. We hypothesize that under such conditions the costs for maintaining the symbioses may outweigh the benefits, ultimately turning the mutualist microbes into parasites, resulting in reduced growth and reproduction of colonized plants. In nature, light availability is often a variable resource due to competition among plant species and, depending on cultivation method, also shows strong variation in agricultural systems [17]. In a few previous studies it was shown that the effects of light limitation on plant growth did not differ between unfertilized plants growing with symbionts and plants growing without symbionts but with additional fertilization [18,19], thereby neglecting the role of nutrient supply by the symbionts as the ultimate benefit of these symbioses for the plant.

In the present study we used lima bean (Phaseolus lunatus), a model plant in chemical ecology and an important food plant [20–24] to better understand the concerted effects of AMF/rhizobial colonization and light availability on vegetative and reproductive traits. Plants were inoculated with rhizobia (R) and AMF and blocks were exposed to two different levels of light availability. Treatments included: no symbiont (R-AMF-), AMF only (R-AMF+), rhizobia only (R+AMF-), and both symbionts (R+AMF+). While effects of light on plant resource allocation patterns (including carbon, nitrogen and phosphate) to either rhizobia [15] or AMF [25–30], have been studied using both empirical and modelling approaches, research on the effects of light upon interactions with multiple bacterial and fungal rhizospheric symbionts simultaneously is limited. However, as plants are frequently colonized by multiple microbial symbionts considering interactive effects among microbes, which may range from competition to cooperation of mutualists, is of high importance [31,32].

This study aims to answer the following specific questions: i) What are the separate and interacting effects of rhizobia and arbuscular mycorrhizal fungi on growth and reproduction of lima bean, and ii) what are the outcomes of these belowground mutualistic interactions regarding plant growth and reproduction when light availability is limited. To our knowledge, our study is among the first to analyze the interactive effects of AMF, rhizobia and light availability, addressing potential costs of these microbes when photosynthesis is limited.

## Materials and Methods

Lima bean (Fabaceae: *Phaseolus lunatus* L.) represents an established model system in plant ecological research due to the simultaneous expression of multiple traits (direct and indirect defenses) affecting the interaction with plant consumers and higher trophic levels [33–35]. Recent studies using the lima bean system further highlight complex bottom-up effects of plant-associated belowground microbial symbionts on above ground food webs and pathogens and thus raise the need for a better understanding of factors driving these belowground associations [22,23]. In this study lima bean plants (cv. Henderson) were grown from surface sterilized seeds (American Meadows Inc., Williston, VT) [26]. Seeds were sterilized by manual agitation in a 10% bleach solution for 2 min, rinsed three times with deionized water and then exposed to an elevated temperature (60°C for 3 h) in an oven. This treatment has been shown to produce sterile plants but does not reduce germination rates in preliminary experiments. Plants were cultivated in a greenhouse with a temperature of 30°C during the light period and at 23°C during the dark period. Relative air humidity was adjusted to 70–80%. Plants were grown in plant-containers of 10 × 10 × 11 cm (width, length, height; one plant per pot) in a 1:1 ratio of low-nutrient seedling potting soil (Fox Farms, Arcata, CA, USA) and washed sand (grain size 0.5–2.0 mm; Quikcrete, GA, USA). The substrate was flushed with 2 L ddH_2_O per kg soil to further reduce nutrient concentration and autoclaved at 121°C for 30 min at a pressure of 1260 mbar. All plants were randomly assigned to one of the four inoculation treatments (R-AMF-; R-AMF+; R+AMF-; R+AMF+) and to one of two light treatments (full light: 600 ± 100 μmol m^-2^s^-1^, 50% shading (shade net): 280 ± 80 μmol m^-2^s^-1^) with a light:dark period of 13:11 h L:D. Plants were watered with autoclaved water as needed and care was taken not to cross-contaminate microbial treatments through splash water during watering. Access water passed through the tables (metal grid top) and was collected in plastic containers under the tables for disposal. Plastic containers were cleaned with bleach once a week.

Every treatment combination had 15 replicates resulting in a total of 120 plants. Plants were inoculated with commercial AMF powder inoculant [Bio Organics^™^, La Pine, Oregon; (*Glomus aggregatum*, *G*. *etunicatum*, *G*. *mosseae*, *G*. *clarum*, *G*. *deserticola*, *G*. *monosporus*, *Gigaspora margarita*, *Paraglomus brasilianum*, *Rhizophagus irregularis*), 10 cc (8 g) per plant; see Millar and Ballhorn [26] for details regarding application. In our previous studies, this inoculum has been shown to form of active mycorrhiza with lima bean and to promote plant growth compared to non-inoculated plants. The rhizobia strain used in our study was isolated from lima bean roots at natural sites in Mexico and identified as *Rhizobium* based on 16S rDNA sequence data [36]. This specific strain represents an efficiently nitrogen-fixing symbiont for lima bean which enhances plant growth as well as the plant’s ability to invest in nitrogen-based defenses [37]. Rhizobia were cultivated in liquid medium (pH 7.0) containing 1 g yeast extract (Amresco), 10 g mannite (Amresco), 800 ml deionized water, and 200 ml soil extract. The soil extract was prepared from 160 g dry, non-fertilized loamy soil (taken from a grass-covered area on the campus of Portland State University) that was suspended in 400 ml deionized water under addition of 0.4 g sodium carbonate (VWR, Radnor, PA) and autoclaved at 121°C for 30 min at a pressure of 1260 mbar. Three days prior to plant inoculation, rhizobia were cultivated at 28°C and 180 rpm on a laboratory shaker (New Brunswick Scientific I26). The bacteria solution was then diluted with autoclaved tap water in a ratio of 1:10 and plants were watered with 100 ml of this solution (>10^7^ cells mL^–1^; determined using a Petroff-Hausser counting chamber). Plants with AMF and rhizobia were inoculated at the same time. Control plants were supplied with autoclaved AMF powder and bacterial media solutions (containing no bacteria) in volumes corresponding to those used in the inoculation treatments while all other parameters remained unchanged.

At the end of the 14 week experiment we evaluated microbial colonization, above and belowground biomass, seed number, and seed viability (S1 Table). Plant tissue was dried in an oven (IncuMax^™^ CV250 Convection Oven, Amerex Instruments, Inc., Lafayette, CA) at 70°C for 5 days. To assess viability of seeds, they were germinated by placing them between wet paper towels in the dark at 25°C. The number of germinating seeds (i.e. development of an intact root) was recorded over a period of 14 days. Seeds that did not germinate after 14 days were considered non-viable.

Percent root-length colonization by AMF was evaluated using the Magnified Intersections Method according to McGonigle et al. [38] after staining. Microscopic observations were conducted using an AmScope FM320 Trinocular Microscope in both 100x and 400x magnification. Rhizobial nodulation (i.e. biomass of rhizobial nodules) was determined per plant after washing soil from roots, removing all visible nodules and drying root nodules at 70°C for 5 days.

### Statistical Analysis

The effects of rhizobia, AMF and light on plant traits were tested as orthogonal factors including all interactions with generalized linear models (GLM). The effects of rhizobia on mycorrhization and vice versa were tested in two-way GLMs with light as additional factor. For number of seeds we assumed poisson-distributed residuals with the log-link function and for mycorrhization rate and seed germination rate we assumed binomial-distributed residual with the logit-link function (both tested against alternative distributions using the Akaike Information Criterion). All analyses were run in SAS 9.2 (Proc Glimmix).

## Results

**Fig 1:**
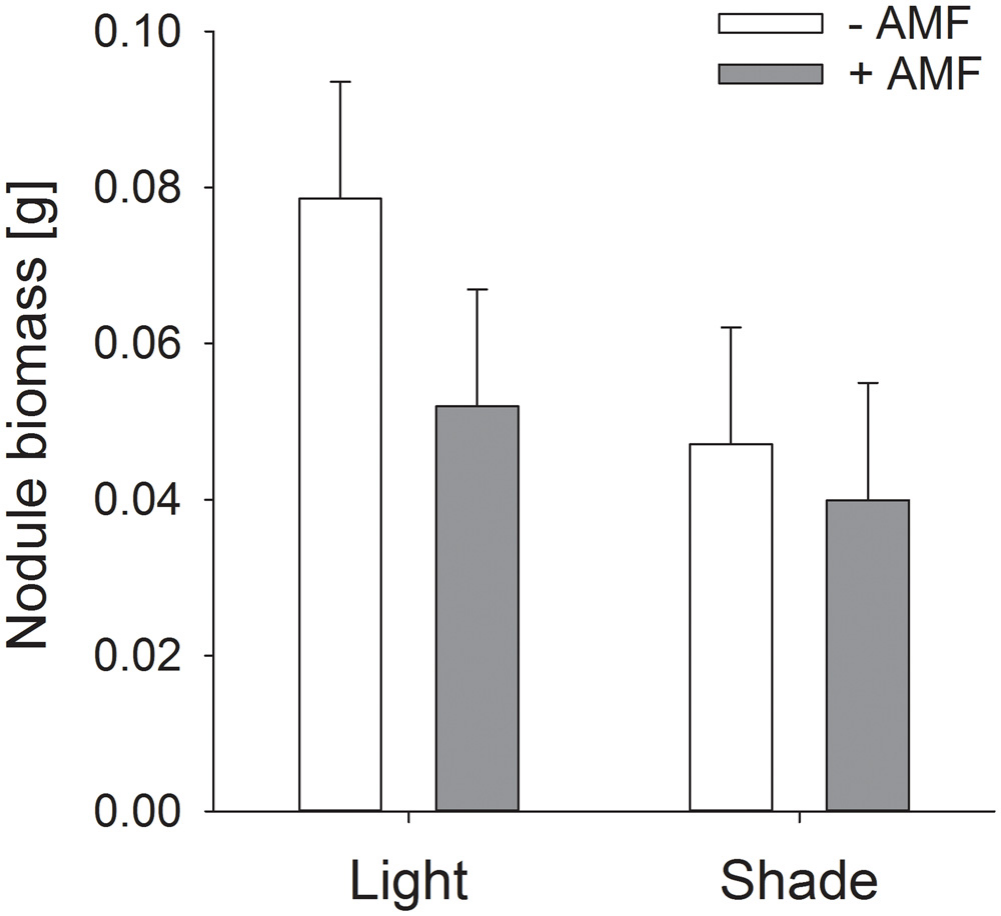
Effects of AMF on nodule biomass of rhizobia-inoculated plants for *P*. *lunatus* under varying light conditions. Bars are means + s.e.

Microscopic analyses revealed successful rhizobial and AMF colonization in inoculated groups, whereas non-inoculated plants showed no rhizobia or AMF. While AMF colonization (overall mean = 14.7%, standard error = 2.7%) was not affected by light availability (*F*_*1*,*56*_ = 1.86, *P* = 0.18), the presence of rhizobia (*F*_*1*,*56*_ = 1.30, *P* = 0.26) or the interaction between both factors (*F*_*1*,*56*_ = 0.47, *P* = 0.49), nodule biomass was reduced 27% by mycorrhization (Fig 1, *F*_*1*,*56*_ = 7.15, *P* = 0.009). The effect of mycorrhization on nodulation tended to be weaker under low-light conditions (- 15%) than under high-light conditions (-33%, mycorrhization x light interaction *F*_*1*,*56*_ = 3.39, *P* = 0.07).

**Fig 2:**
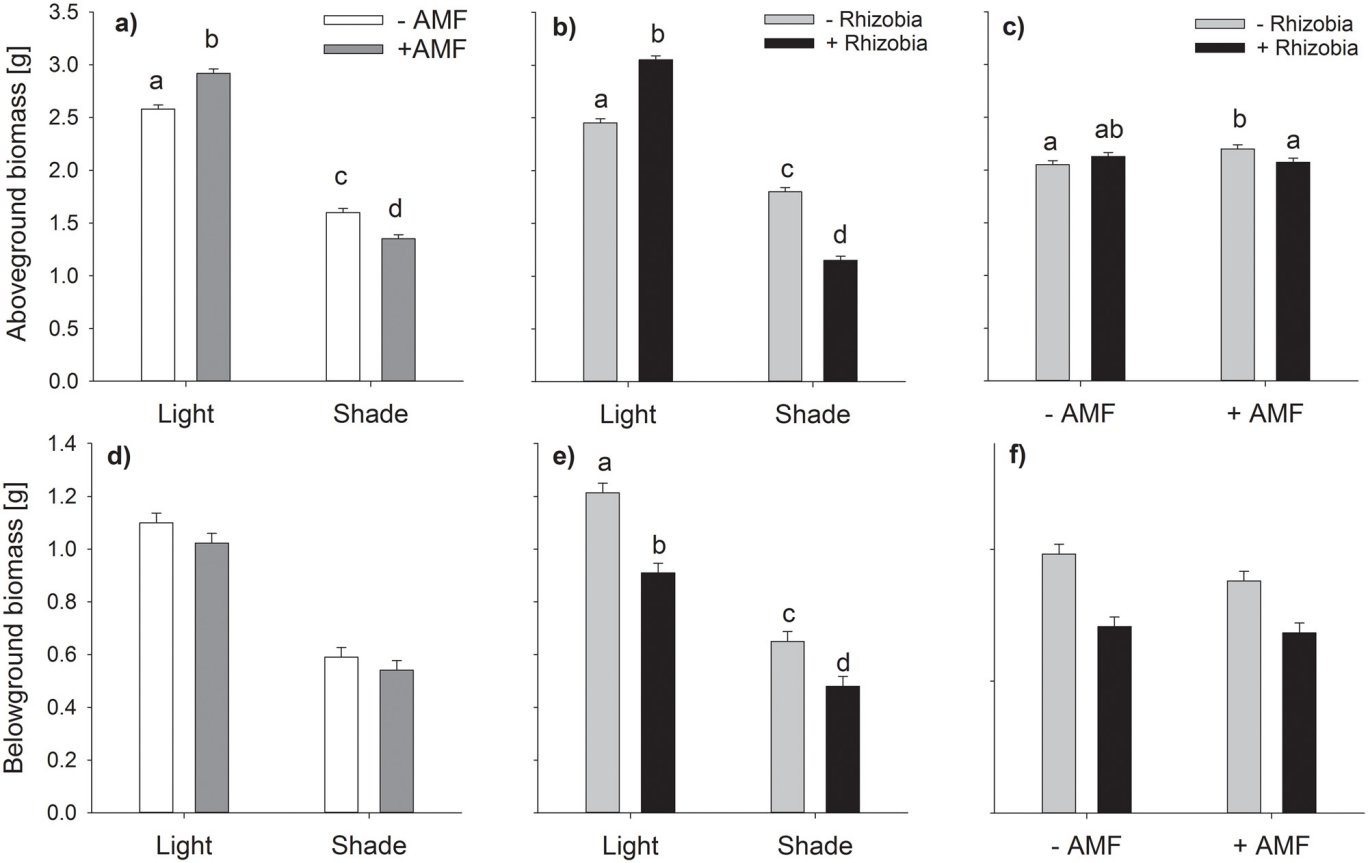
Interacting effects of varying light conditions, AMF and rhizobia on aboveground (a, b, c) and belowground biomass (d, e, f). Bars are means + s.e. Different letters indicate statistically different means following Tukey’s post-hoc test (*P*<0.05). Only significant interactions are given as means of the corresponding factorial combinations, thus (a) and (d) include all rhizobial treatments, (b) and (e) include all mycorrhizal treatments and (c) and (f) include all light treatments. Only tests with a significant interaction between two factors are followed by a post-hoc test.

Aboveground biomass was an average of 86% higher under full light than shade. The effects of AMF and rhizobia strongly depended on the light treatments. The positive effects of both symbionts under full light turned into negative effects for the shade plants (Table 1, Fig 2a and 2b, S1 Table). Plants inoculated with both AMF and rhizobia exhibited a 43% increase of mean aboveground biomass relative to non-inoculated plants under full light, whereas mean aboveground biomass of fully inoculated shade plants decreased by 47% relative to AMF and rhizobia-free plants. AMF and rhizobia showed further interacting effects on aboveground biomass independent of light treatment, resulting in an AMF-induced 8% increase of aboveground biomass only for plants without rhizobia (Table 1, Fig 2c, S1 Table).

**Table 1:** GLM-results of the effects of rhizobial nodulation, mycorrhizal colonization and varying light conditions on vegetative traits of *P*. *lunatus*. Significant effects are shown in bold.

	Aboveground Biomass		Belowground Biomass		Total Biomass		Shoot:Root	
	*F*_*1*,*112*_	*P*	*F*_*1*,*112*_	*P*	*F*_*1*,*112*_	*P*	*F*_*1*,*112*_	*P*
Rhizobia (R)	0.46	0.50	**40.96**	**<0.001**	**18.85**	**<0.001**	**15.90**	**<0.001**
Mycorrhiza (AMF)	1.62	0.21	2.93	0.09	0.05	0.82	0.48	0.49
Light (L)	**1049.3**	**<0.001**	**180.39**	**<0.001**	**851.48**	**<0.001**	0.46	0.49
R × AMF	**6.91**	**0.01**	1.17	0.28	1.09	0.30	2.26	0.14
R × L	**248.76**	**<0.001**	3.25	0.07	**83.21**	**<0.001**	**42.21**	**<0.001**
AMF × L	**56.96**	**<0.001**	0.13	0.72	**21.82**	**<0.001**	**5.43**	**0.02**
R × AMF × L	2.08	0.15	1.37	0.24	0.05	0.83	0.28	0.60

**Fig 3:**
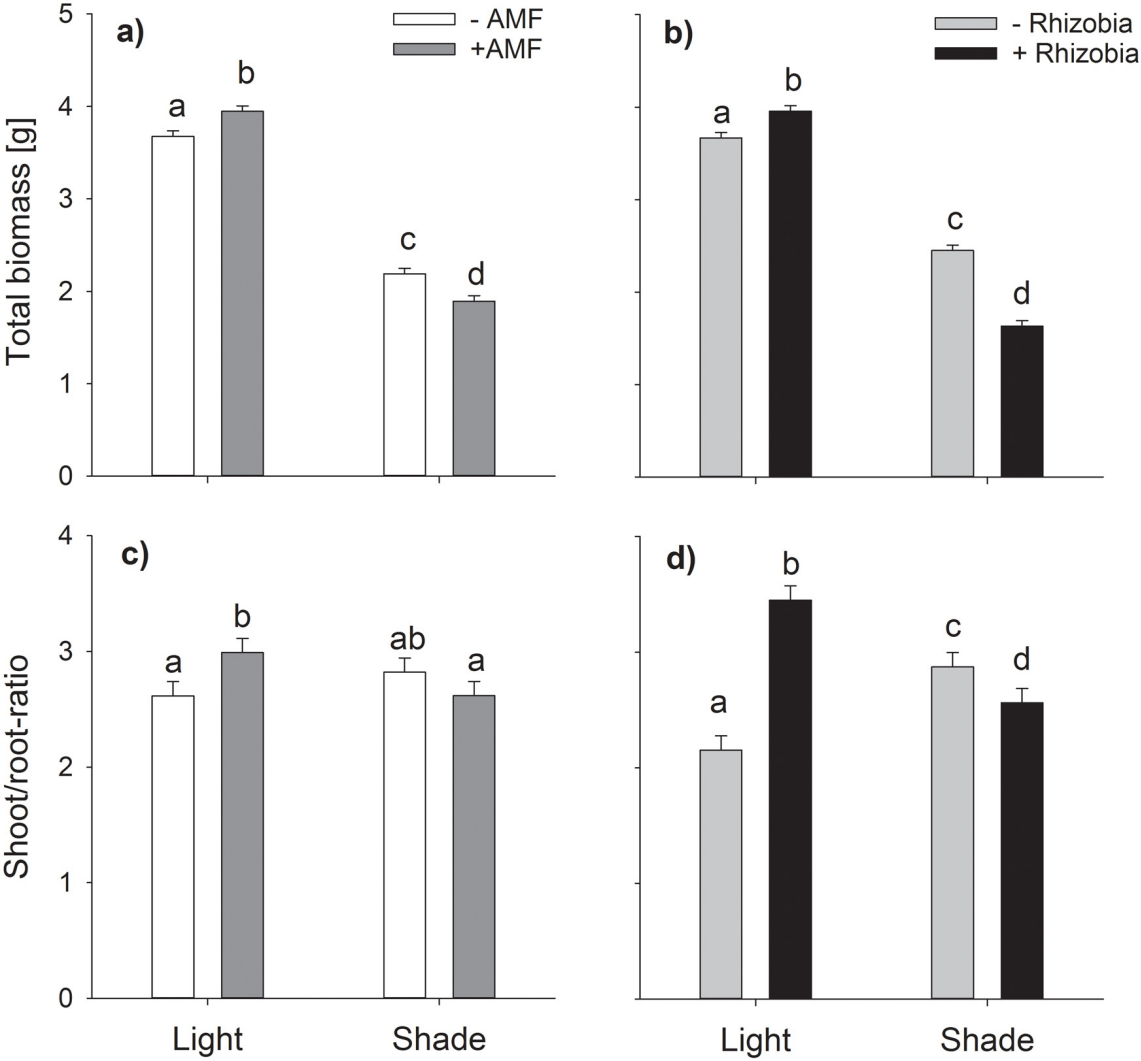
Interacting effects of varying light conditions and AMF colonization on total biomass (a) and shoot/root-ratio (c) and interacting effects of varying light conditions and rhizobial nodulation on total biomass (b) and shoot/root-ratio (d). Bars are means + s.e. Only significant interactions are given as means of the corresponding factorial combinations, thus (a) and (c) include all rhizobial treatments and (b) and (d) include all mycorrhizal treatments. Different letters indicate statistically different means following Tukey’s post-hoc test (*P*<0.05).

Belowground biomass was unaffected by mycorrhization but was decreased by 47% under shaded conditions independent of inoculation with mycorrhiza, and by 28% in rhizobia treatments independent of light condition (Table 1, Fig 2d and 2e, S1 Table). The negative effect of rhizobia was independent on mycorrhization (no significant interaction, Table 1, Fig 2f, S1 Table). Similar to aboveground biomass, total plant biomass was 87% higher under full light. However, inoculation decreased belowground biomass under both light treatments, with rhizobia accounting for a greater proportion of the decrease (Table 1, Fig 3a and 3b, S1 Table). Shoot/root-ratio showed no general effect of the light treatment but a higher relative investment into aboveground biomass with AMF and rhizobia under full light, whereas under low-light conditions rhizobia decreased shoot/root-ratio (Table 1, Fig 3c and 3d, S1 Table).

**Fig 4:**
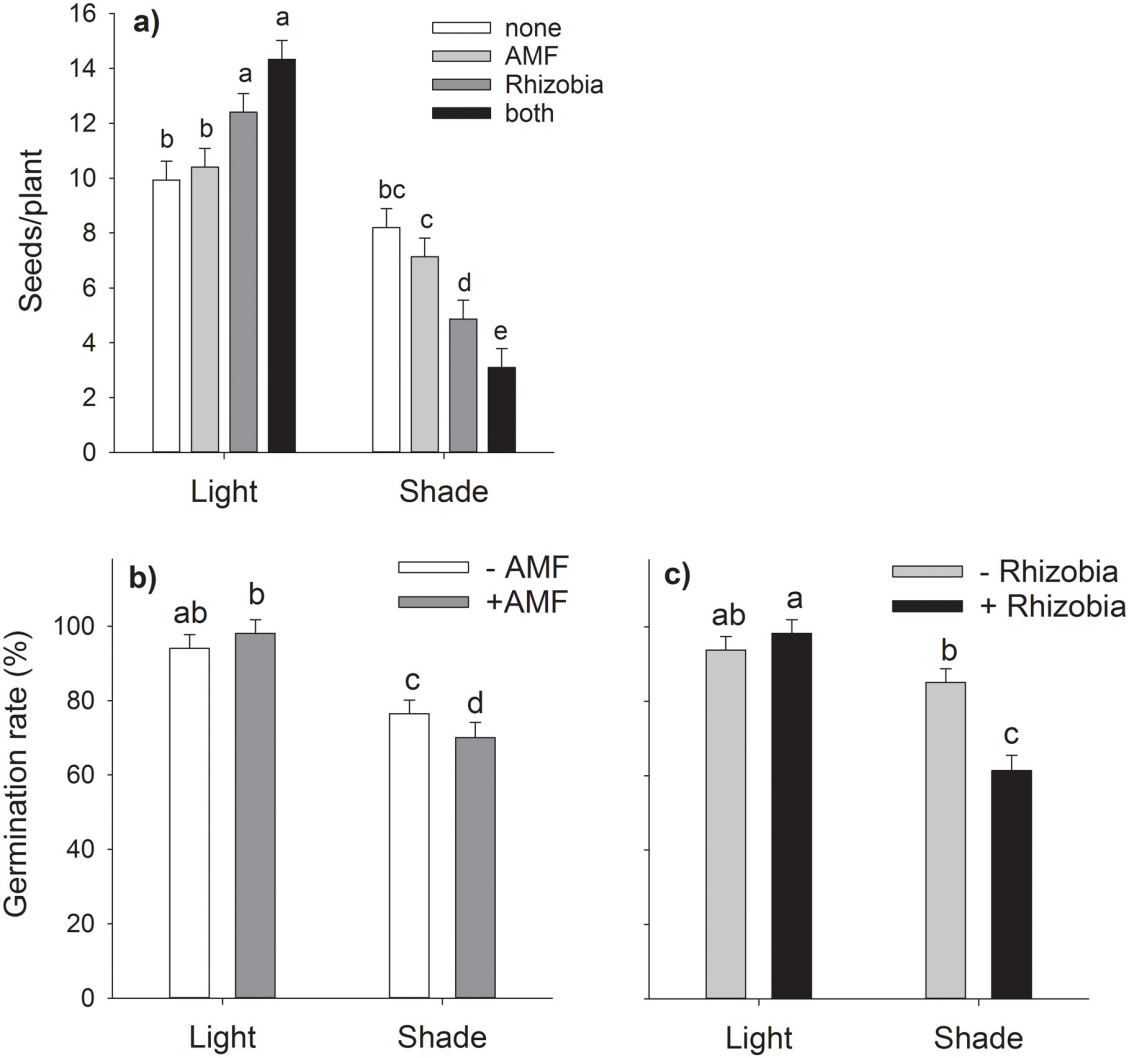
Interacting effects of varying light conditions, AMF and rhizobia on the number of produced seeds per plant (a) and germination rate (b,c). Bars are means + s.e. Only significant interactions are given as means of the corresponding factorial combinations, thus (a) and (d) include all rhizobial treatments, (b) and (e) include all mycorrhizal treatments and (c) and (f) include all light treatments. Different letters indicate statistically different means following Tukey’s post-hoc test (*P*<0.05).

The number of seeds produced per plant was affected by a three-way interaction between the factors light, AMF and rhizobia. The overall positive effect of light was further increased by the symbiosis with rhizobia but not significantly by AMF. For plants under low-light, rhizobia strongly decreased the number of seeds and this effect was further exacerbated by AMF, whereas AMF alone had no significant effect (Table 2, Fig 4a, S1 Table).

**Table 2:** GLM-results of the effects of rhizobial nodulation, mycorrhizal colonization and varying light conditions on generative traits of *P*. *lunatus* and the mutual influence between both symbionts in *P*. *lunatus*. Significant effects are shown in bold.

	Total Seeds		Germination [%]	
	*F*_*1*,*112*_	*P*	*F*_*1*,*106*_	*P*
Rhizobia (R)	**17.13**	**< 0.001**	0.02	0.89
Mycorrhiza (AMF)	**7.49**	**0.07**	0.15	0.70
Light (L)	**136.73**	**<0.001**	**48.51**	**<0.001**
R × AMF	**4.41**	**0.04**	0.11	0.74
R × L	**61.27**	**<0.001**	**19.34**	**<0.001**
AMF × L	**16.26**	**<0.001**	**7.06**	**0.009**
R × AMF × L	**7.69**	**0.006**	2.63	0.11

The effects of the light treatment on the germination rate of seeds were again dependent on the presence of root symbionts. The negative effect of shading was more pronounced for plants growing with both AMF and rhizobia (Table 2, Fig 4b and 4c, S1 Table).

## Discussion

In our study we quantitatively analyzed effects of light availability on the tripartite symbiosis of mycorrhizal fungi (AMF), rhizobia, and lima bean. Our results showed enhancing effects on growth and reproduction by belowground symbionts under full light, but reduced plant development and reproduction in inoculated plants under shaded conditions. Moreover, nodulation of plants was reduced by AMF resulting in interacting effects of both types of symbionts on growth and reproduction. Interacting effects of rhizobia and AMF on plant growth are sometimes suggested to be rather uncommon [39,40], however, we could demonstrate an antagonistically interacting effect on aboveground biomass. This antagonistic effect was also obvious for the reproduction of lima bean but only under conditions of light limitation. This implies that light availability mediates the outcome of a dual infection with different root symbionts.

Carbon requirements by root-associated microbial mutualists are thought to be easily compensated through sink stimulation (up-regulation) of photosynthesis [13,26]. Our study shows that even though sink stimulation may be an efficient strategy to compensate for photosynthate losses to microbial symbionts under full light, the situation can be fundamentally different when light is limited. Under such conditions, mutualistic microbes may act as parasites that exploit resources and reduce host fitness [15,41,42]. As spatial and temporal variation in light availability are ubiquitous in nature [17], as are plant associations with multiple rhizospheric microbes, our findings suggest that light variation represents a widely overlooked key factor determining the outcome of plant-microbe interactions.

Inquiry into the mutualism/parasitism continuum offers insight into the delicate coevolution of plants and their belowground symbionts. Kiers and Denison [43] have shown that rhizobial mutualisms are maintained by host sanctioning of “cheating” genotypes while AMF mutualisms are maintained by reciprocal rewards between genotypes of hosts and AMF that allocate a greater amount of nutrient or photosynthate to the more generous partner. Though plants have relatively fine control over these mechanisms via selective partitioning within root systems when multiple microbial genotypes are present [43], an overall change in an abiotic condition such as light is likely to have a disruptive effect on the symbiosis. Whether either sanctioning or reciprocal rewards are at play, mutualistic cooperation also favors mutualist fitness, thus we predict a destabilization of mutualisms under unfavorable abiotic conditions that induce a symbiotic lifestyle switch. Our study shows that light availability is a key factor in determining the threshold between mutualist and parasite in such interactions.

Legumes, rhizobia, and mycorrhizal fungi usually form a tripartite symbiosis, and legume performance may therefore be affected by potential interactions between both symbionts. In our study, nodulation was reduced for plants grown with AMF. Nutrient provisions by one microbial symbiont may reduce the provisional benefits of the other. Multiple studies have addressed reduced benefits of rhizospheric symbioses in the presence of non-limiting resources [19,44,45]. The reduced nodulation we observed in the presence of AMF might also be explained by competition for colonization sites, even if evidence for this is controversial [43]. AMF are sometimes described as the dominant symbiont due to the prioritization of phosphorous over N provision to both host and rhizobia during AMF colonization [46]. However, we found that this effect tended to be more obvious under full light, whereas under shaded conditions nodulation was generally weak regardless of the status of AMF infection, suggesting competition for photosynthates. This interaction was also reflected by an antagonistic effect of both symbionts on biomass and seed production under light limitation, which is in dramatic contrast to synergistic effects under full light reported in the present study and others [46]. Differences in abiotic conditions (e.g. light availability) may therefore be interpreted as an important mediator of such mutualistic relationships.

Many questions remain regarding whether all legumes may exhibit similar decreases in fitness parameters due to AMF and rhizobial colonization under light-limited conditions. A better understanding of these interactions is of high relevance. As of 2003, grain and forage legumes represented 27% of all primary agricultural production [20]. In natural ecosystems, ranging from forests to grasslands, microbial associations with legumes have been shown to be responsible for the provision of the majority of available nitrogen [2,20]. Considering the ubiquity of the highlighted symbiotic associations and the economic importance of legumes, conditional symbiotic lifestyle switching (mutualist to parasite) has significant ramifications regarding the stability of mutualisms and the productivity of agro-ecological systems.

It further remains to be tested whether the effects we report here hold true under natural conditions. While we used a natural rhizobia strain derived from a wild lima bean population, the AMF inoculum we used in the present study represents a commercial product. Thus, it likely contains AMF species and strains that form beneficial interactions with a broad range of different host plant species but may not interact with lima bean plants in nature. Also, using an inoculum composed of various fungi leaves the question which of the strains actually forms beneficial mycorrhiza and causes in the observed effects. Alternatively, several AMF species may co-colonize plant roots simultaneously and thus can introduce uncontrolled variation into the experimental system due to variable synergistic effects or competition among fungi but also between fungi and rhizobia. However, as we observed consistent effects of AMF and rhizobial inoculation, as well as for plants inoculated with both root symbionts, our statements on the effect of light limitation on this experimental tripartite interaction seem justified. Whether or not such effects determine the outcome of diverse tripartite plant-AMF-rhizobia interactions in natural ecosystems remains to be tested.

## Supporting Information

S1 Table Raw data on effects of light availability (L+ = full light, L- = 50% light), rhizobial (R+ = with rhizobia, R- = no rhizobia) and mycorrhizal (M+ = with AMF fungi, M- = no AMF fungi) colonization on production and viability of seeds in lima bean (*Phaseolus lunatus*). (DOCX)
