# Supplementary material for: Friend or Foe—Light Availability Determines the Relationship between Mycorrhizal Fungi, Rhizobia and Lima Bean (Phaseolus lunatus L.)
Source: PLoS One. 2016 May 2;11(5):e0154116. doi: 10.1371/journal.pone.0154116 (PMC4852939; doi:10.1371/journal.pone.0154116)
Supplement: S1 Table — (DOCX) [file pone.0154116.s001.docx]

**Table S1. Raw data on effects of light availability (L+ = full light, L- = 50% light), rhizobial (R+ = with rhizobia, R- = no rhizobia) and mycorrhizal (M+ = with AMF fungi, M- = no AMF fungi) colonization on production and viability of seeds in lima bean (*Phaseolus lunatus*).**

| Treatment | Aboveground biomass  [g DW] | Belowground biomass  [g DW] | AMF colonization  [%] | Nodule biomass  [g DW] | Seeds  [#] | Viability of seeds  (# non-germinating) |
| --- | --- | --- | --- | --- | --- | --- |
|  |  |  |  |  |  |  |
| L+ R+ M+ | 3.2 | 0.9 | 15 | 0.034 | 15 | 0 |
| L+ R+ M+ | 3.5 | 1 | 22 | 0.012 | 12 | 0 |
| L+ R+ M+ | 3.1 | 0.9 | 12 | 0.014 | 13 | 1 |
| L+ R+ M+ | 3.4 | 0.7 | 13 | 0.037 | 19 | 0 |
| L+ R+ M+ | 2.8 | 0.9 | 14 | 0.03 | 14 | 0 |
| L+ R+ M+ | 3.3 | 1.1 | 14 | 0.041 | 20 | 0 |
| L+ R+ M+ | 2.7 | 1.1 | 12 | 0.001 | 9 | 1 |
| L+ R+ M+ | 2.8 | 0.9 | 20 | 0.03 | 15 | 0 |
| L+ R+ M+ | 3.1 | 0.7 | 9 | 0.01 | 12 | 0 |
| L+ R+ M+ | 3.2 | 0.9 | 11 | 0.012 | 13 | 0 |
| L+ R+ M+ | 2.9 | 1.1 | 12 | 0.001 | 10 | 0 |
| L+ R+ M+ | 3.1 | 0.8 | 13 | 0.45 | 13 | 0 |
| L+ R+ M+ | 3.1 | 0.8 | 14 | 0.021 | 12 | 0 |
| L+ R+ M+ | 3.3 | 1 | 15 | 0.051 | 22 | 0 |
| L+ R+ M+ | 3.6 | 0.9 | 21 | 0.035 | 16 | 0 |
|  |  |  |  |  |  |  |
| L+ R- M - | 2.1 | 1 | 0 | 0 | 13 | 1 |
| L+ R- M - | 2.4 | 1.5 | 0 | 0 | 5 | 0 |
| L+ R- M - | 2.4 | 1.2 | 0 | 0 | 12 | 0 |
| L+ R- M - | 2.3 | 0.9 | 0 | 0 | 7 | 3 |
| L+ R- M - | 2 | 0.7 | 0 | 0 | 13 | 1 |
| L+ R- M - | 1.8 | 0.9 | 0 | 0 | 5 | 0 |
| L+ R- M - | 2 | 1.1 | 0 | 0 | 11 | 1 |
| L+ R- M - | 2.1 | 1.4 | 0 | 0 | 10 | 0 |
| L+ R- M - | 2.5 | 1.9 | 0 | 0 | 11 | 0 |
| L+ R- M - | 2.4 | 1.7 | 0 | 0 | 8 | 0 |
| L+ R- M - | 2.5 | 1.8 | 0 | 0 | 11 | 2 |
| L+ R- M - | 1.8 | 1.2 | 0 | 0 | 13 | 0 |
| L+ R- M - | 1.9 | 1.4 | 0 | 0 | 9 | 3 |
| L+ R- M - | 2.5 | 1.9 | 0 | 0 | 11 | 2 |
| L+ R- M - | 2.3 | 0.8 | 0 | 0 | 10 | 1 |
|  |  |  |  |  |  |  |
| L+ R- M + | 2.9 | 0.9 | 18 | 0 | 11 | 0 |
| L+ R- M + | 2.8 | 1 | 12 | 0 | 10 | 0 |
| L+ R- M + | 2.8 | 1.4 | 13 | 0 | 9 | 0 |
| L+ R- M + | 2.5 | 1.3 | 14 | 0 | 12 | 1 |
| L+ R- M + | 2.6 | 1.3 | 19 | 0 | 11 | 0 |
| L+ R- M + | 2.8 | 1.4 | 22 | 0 | 13 | 0 |
| L+ R- M + | 2.7 | 1.1 | 21 | 0 | 14 | 1 |
| L+ R- M + | 2.7 | 0.9 | 13 | 0 | 11 | 1 |
| L+ R- M + | 2.6 | 1 | 15 | 0 | 10 | 0 |
| L+ R- M + | 2.9 | 0.9 | 16 | 0 | 9 | 0 |
| L+ R- M + | 2.8 | 1.4 | 20 | 0 | 10 | 0 |
| L+ R- M + | 2.7 | 1 | 19 | 0 | 8 | 0 |
| L+ R- M + | 2.4 | 0.9 | 15 | 0 | 7 | 1 |
| L+ R- M + | 2.6 | 1.4 | 12 | 0 | 10 | 0 |
| L+ R- M + | 2.8 | 1.1 | 14 | 0 | 11 | 0 |
|  |  |  |  |  |  |  |
| L+ R+ M - | 3 | 0.9 | 0 | 0.044 | 10 | 1 |
| L+ R+ M - | 3.3 | 1.1 | 0 | 0.064 | 12 | 0 |
| L+ R+ M - | 3 | 1 | 0 | 0.108 | 18 | 0 |
| L+ R+ M - | 3.2 | 0.9 | 0 | 0.062 | 12 | 0 |
| L+ R+ M - | 3 | 0.8 | 0 | 0.04 | 11 | 0 |
| L+ R+ M - | 2.9 | 0.5 | 0 | 0.054 | 13 | 0 |
| L+ R+ M - | 3.1 | 0.9 | 0 | 0.076 | 9 | 0 |
| L+ R+ M - | 3.2 | 0.7 | 0 | 0.068 | 8 | 0 |
| L+ R+ M - | 2.5 | 0.9 | 0 | 0.046 | 10 | 0 |
| L+ R+ M - | 2.9 | 1.1 | 0 | 0.082 | 9 | 1 |
| L+ R+ M - | 3 | 1.1 | 0 | 0.138 | 17 | 0 |
| L+ R+ M - | 3.1 | 0.9 | 0 | 0.11 | 20 | 0 |
| L+ R+ M - | 2.9 | 0.8 | 0 | 0.098 | 14 | 0 |
| L+ R+ M - | 2.3 | 0.9 | 0 | 0.1142 | 14 | 0 |
| L+ R+ M - | 2.9 | 1.1 | 0 | 0.074 | 9 | 1 |
|  |  |  |  |  |  |  |
| L- R- M - | 2 | 0.7 | 0 | 0 | 9 | 1 |
| L- R- M - | 2 | 0.6 | 0 | 0 | 10 | 0 |
| L- R- M - | 1.7 | 0.8 | 0 | 0 | 8 | 0 |
| L- R- M - | 1.8 | 0.7 | 0 | 0 | 7 | 0 |
| L- R- M - | 2 | 0.7 | 0 | 0 | 6 | 2 |
| L- R- M - | 1.9 | 0.5 | 0 | 0 | 6 | 0 |
| L- R- M - | 1.9 | 0.6 | 0 | 0 | 9 | 0 |
| L- R- M - | 1.8 | 0.5 | 0 | 0 | 5 | 2 |
| L- R- M - | 1.8 | 0.7 | 0 | 0 | 9 | 1 |
| L- R- M - | 2 | 0.6 | 0 | 0 | 12 | 0 |
| L- R- M - | 2.2 | 0.9 | 0 | 0 | 11 | 0 |
| L- R- M - | 1.6 | 0.6 | 0 | 0 | 7 | 0 |
| L- R- M - | 2 | 0.9 | 0 | 0 | 6 | 0 |
| L- R- M - | 1.9 | 0.8 | 0 | 0 | 10 | 0 |
| L- R- M - | 1.9 | 0.5 | 0 | 0 | 8 | 1 |
|  |  |  |  |  |  |  |
| L- R- M + | 2 | 0.8 | 12 | 0 | 6 | 0 |
| L- R- M + | 1.9 | 0.5 | 14 | 0 | 5 | 4 |
| L- R- M + | 1.8 | 0.7 | 17 | 0 | 8 | 1 |
| L- R- M + | 1.3 | 0.6 | 18 | 0 | 9 | 0 |
| L- R- M + | 1.5 | 0.5 | 21 | 0 | 7 | 3 |
| L- R- M + | 1.6 | 0.8 | 20 | 0 | 7 | 4 |
| L- R- M + | 2.1 | 0.9 | 13 | 0 | 7 | 0 |
| L- R- M + | 1.8 | 0.6 | 12 | 0 | 4 | 2 |
| L- R- M + | 2.1 | 0.5 | 10 | 0 | 6 | 2 |
| L- R- M + | 1.8 | 0.7 | 9 | 0 | 5 | 2 |
| L- R- M + | 1.3 | 0.6 | 13 | 0 | 7 | 0 |
| L- R- M + | 1.4 | 0.4 | 15 | 0 | 12 | 1 |
| L- R- M + | 1.3 | 0.5 | 17 | 0 | 8 | 0 |
| L- R- M + | 1.5 | 0.4 | 12 | 0 | 7 | 1 |
| L- R- M + | 2.1 | 0.9 | 11 | 0 | 9 | 0 |
|  |  |  |  |  |  |  |
| L- R+ M - | 1.2 | 0.4 | 0 | 0.037 | 7 | 2 |
| L- R+ M - | 1.4 | 0.4 | 0 | 0.044 | 4 | 1 |
| L- R+ M - | 1.4 | 0.3 | 0 | 0.054 | 1 | 1 |
| L- R+ M - | 1.5 | 0.5 | 0 | 0.065 | 7 | 2 |
| L- R+ M - | 1.4 | 0.4 | 0 | 0.05 | 3 | 2 |
| L- R+ M - | 1.4 | 0.5 | 0 | 0.03 | 7 | 0 |
| L- R+ M - | 1.1 | 0.5 | 0 | 0.03 | 9 | 3 |
| L- R+ M - | 1.2 | 0.6 | 0 | 0.046 | 4 | 1 |
| L- R+ M - | 1.4 | 0.7 | 0 | 0.065 | 0 | 0 |
| L- R+ M - | 1.2 | 0.4 | 0 | 0.032 | 4 | 0 |
| L- R+ M - | 1.2 | 0.4 | 0 | 0.041 | 7 | 0 |
| L- R+ M - | 1.1 | 0.5 | 0 | 0.058 | 8 | 2 |
| L- R+ M - | 1.2 | 0.6 | 0 | 0.063 | 1 | 1 |
| L- R+ M - | 1.4 | 0.7 | 0 | 0.032 | 5 | 3 |
| L- R+ M - | 1.4 | 0.7 | 0 | 0.059 | 6 | 4 |
|  |  |  |  |  |  |  |
| L- R+ M + | 1.1 | 0.3 | 12 | 0.061 | 2 | 1 |
| L- R+ M + | 1.1 | 0.5 | 13 | 0.025 | 1 | 0 |
| L- R+ M + | 1.2 | 0.6 | 7 | 0.03 | 1 | 1 |
| L- R+ M + | 0.9 | 0.7 | 11 | 0.03 | 4 | 1 |
| L- R+ M + | 1.1 | 0.4 | 21 | 0.04 | 6 | 2 |
| L- R+ M + | 1 | 0.3 | 16 | 0.033 | 0 | 0 |
| L- R+ M + | 1.1 | 0.5 | 18 | 0.046 | 2 | 2 |
| L- R+ M + | 0.8 | 0.4 | 12 | 0.032 | 2 | 0 |
| L- R+ M + | 1.1 | 0.3 | 11 | 0.041 | 0 | 0 |
| L- R+ M + | 0.8 | 0.5 | 9 | 0.058 | 0 | 0 |
| L- R+ M + | 0.9 | 0.5 | 8 | 0.043 | 4 | 0 |
| L- R+ M + | 1.1 | 0.6 | 15 | 0.032 | 5 | 2 |
| L- R+ M + | 0.8 | 0.5 | 15 | 0.044 | 0 | 0 |
| L- R+ M + | 1.1 | 0.4 | 19 | 0.034 | 4 | 1 |
| L- R+ M + | 1 | 0.3 | 21 | 0.05 | 0 | 0 |
